# Cognitive Function in Primary Sjögren’s Syndrome: A Systematic Review

**DOI:** 10.3390/brainsci9040085

**Published:** 2019-04-15

**Authors:** Ciro Manzo, Eva Martinez-Suarez, Melek Kechida, Marco Isetta, Jordi Serra-Mestres

**Affiliations:** 1 Rheumatologic Outpatient Clinic and Gerontorheumatology Service, “Mariano Lauro” Hospital, 80065 Sant’Agnello, Italy; 2 Center for Cognitive Diseases and Dementias, Azienda Sanitaria Locale Napoli 3 sud, 80038 Pomigliano d’Arco, Italy; 3 Geriatrician, Hospital de Mataró (Barcelona), 08304 Mataró, Barcelona, Spain; emartinezsu@csdm.cat; 4 Internal Medicine and Endocrinology Department, Fattouma Bourguiba University Hospital, University of Monastir, 5000 Monastir, Tunisia; kechida_mel_lek@hotmail.com; 5 Library and Knowledge Services, Central and North West London NHS Foundation Trust, London UB8 3NN, UK; marco.isetta@nhs.net; 6 Department of Old Age Psychiatry, Central and North West London NHS Foundation Trust, London UB8 3NN, UK; jordi.serra-mestres@nhs.net

**Keywords:** primary Sjögren’s syndrome, cognitive functions, mild cognitive impairment, brain fog, dementia, fatigue, systematic review

## Abstract

Background: Cognitive disorders are reported to be common in patients with primary Sjogren’s syndrome (pSS). In some cases, they are the first clinical manifestation, preceding the diagnosis of pSS by two years on average. Aim: A systematic review was conducted to explore cognitive impairment in pSS, with reference to diagnostic methods and their relationship with laboratory data and clinical manifestations. Materials and Methods: According to the PRISMA 2009 checklist, we carried out a comprehensive literature search in the three main bibliographic databases: MEDLINE, EMBASE, and PsycINFO (NICE HDAS interface). The following main search terms were used: primary Sjogren syndrome, neurological manifestations, fatigue, cognitive functions, psychiatric manifestations, mild cognitive impairment, dementia, and neurocognitive disorder. The search was made on 14 September, 2018. References from all selected studies were also examined. Inclusion criteria were: all studies and case-reports published in any language from 2002 that assessed the association of pSS (according to classification criteria proposed by the 2002 American/European collaborative group (AECG)) with all types of cognitive impairment (including dementia). Exclusion criteria were: reviews, abstracts, secondary Sjögren’s syndrome (SS), and all articles in which other classification criteria were used. Results: The initial search yielded 352 articles, of which 253 were excluded based on the title and abstract review. A total of 54 articles underwent a full-length review, and 32 articles were excluded. Data were extracted from 18 studies and three case-reports involving a total of 6196 participants. In most cases, cognitive dysfunction was a brain fog or a mild cognitive impairment (MCI). Occasionally, an autoimmune dementia was present. The relationship between pSS and degenerative dementias, such as Alzheimer’s disease (AD), was a controversial issue, even if some investigators hypothesized that pSS could be a risk factor. Several unmet needs were highlighted. First, some of the included studies had not reported the severity of pSS; hence, few correlations between disease severity and cognitive function were possible. Secondly, the evaluation of the pathogenetic role of comorbid diseases was often absent. The lack of information on the type of dementia represented a third critical point in the majority of the included studies. Conclusions: This systematic review confirmed that adequate studies on cognitive function in pSS are scarce, mostly performed on small-sized samples, and often conflicting. The routine assessment of cognitive function in patients with pSS seems advisable and it will help to elucidate some of the unmet needs highlighted by this review in future appropriately designed studies.

## 1. Introduction

### 1.1. Rationale

Sjögren’s syndrome (SS) is one of the most common connective tissue diseases. It typically presents with a lymphocytic infiltration of exocrine glands leading to sicca syndrome. SS can be present as primary SS (pSS), which is an entity by itself without an underlying autoimmune condition, or secondary SS (sSS) [1,2,3]. At least one-third of patients with pSS can present with extraglandular involvement, with neurological dysfunction being one of the most frequent conditions [4,5]. The rate of neurological symptoms in patients with pSS has been reported to range from 8.5% to 70%. Such a disparate range is a likely consequence of the use of different sets of diagnostic criteria for pSS and of different definitions of neurological syndromes. However, the diagnostic set is important, as there is a greater availability of evaluations of neurological function in patients hospitalized on neurology wards, compared with rheumatology wards [6,7]. The most frequent neurological complication of pSS is peripheral neuropathy, especially sensory polyneuropathy. Central nervous system (CNS) involvement is much less common and has been reported in less than 25% of patients. Simultaneous involvement of the peripheral and the central nervous systems is also possible [8]. In 25–60% of cases, neurological symptoms are the first clinical manifestation and can precede the diagnosis of pSS on an average of two years [9]. Cognitive disorders are reported to be common. Many patients reported mild or minimal subjective cognitive difficulties, often referred to as the so-called “brain-fog”. Brain fog represents a wide range of subjectively experienced cognitive difficulties, such as forgetfulness, memory lapses, mental confusion, reduced verbal fluency, and diminished ability to concentrate more severely in the presence of distractors or competing stimuli [10,11]. Sometimes, cognitive impairment has been thought to be a consequence of other manifestations of pSS, such as fatigue. Fatigue can be defined as an enduring, subjective sensation of generalized tiredness or exhaustion. Unlike weakness, fatigue can be alleviated by periods of rest. Most patients with rheumatic diseases complain of chronic fatigue [12]. Moreover, the relationship between pSS and the dementias, especially Alzheimer’s disease (AD), must be better assessed.

### 1.2. Objectives

A systematic review was conducted to explore cognitive impairment in pSS, with emphasis on diagnostic methods and their relationship with laboratory data and with clinical manifestations. The relationship between pSS and dementias and the role of fatigue were the review’s main focus.

## 2. Materials and Methods

This systematic review was conducted and reported according to the Preferred Reporting Items for Systematic Reviews and Meta-Analyses (PRISMA) guidelines. No review protocol exists.

### 2.1. Search Strategy

One author of this study (Isetta, M.) carried out a comprehensive literature search in the three main bibliographic databases: MEDLINE, EMBASE, and PsycINFO (NICE HDAS interface). The following main search terms were used: primary Sjogren syndrome, neurological manifestations, fatigue, cognitive functions, psychiatric manifestations, mild cognitive impairment, dementia, and neurocognitive disorder. The search was made on 14 September, 2018. References from all of the selected studies were also examined. In accordance with the PRISMA 2009 checklist [13], the full search terms used, together with the results of the searches in one of the three databases (MEDLINE), are detailed in the *Supplemental material and methods* section.

### 2.2. Inclusion Criteria

This review included all studies and case-reports published in any language from 2002 that assessed the association of pSS (according to classification criteria proposed by the 2002 American/European collaborative group (AECG)) with all types of cognitive impairment (including dementia). According to these criteria, the diagnosis of pSS can be made by fulfilling four out of six items. Two items include subjective xerophtalmia and xerostomia; two items include objective evidence of keratoconjunctivitis sicca and xerostomia; two items include a positive minor salivary gland biopsy with a focus score of ≥1/4 mm^2^ or the presence of anti-Ro/SSA or anti-La/SSB antibodies [14].

In 2012, new criteria were proposed by the Sjögren’s International Collaborative Clinical Alliances Cohort [15] and then approved in 2016 by the American College of Rheumatology (ACR) and by the European League against Rheumatism (EULAR). These classification criteria are based on the weighted sum of five items: anti-SSA/Ro antibody positivity and focal lymphocytic sialadenitis with a focus score of ≥1 foci/4 mm^2^, each scoring 3; an abnormal ocular staining score of ≥5 (or van Bijsterveld score of ≥4); a Schirmer’s test result of ≤5 mm/5 min; and an unstimulated salivary flow rate of ≤0.1 mL/min, each scoring 1. Individuals with signs or symptoms suggestive of SS, or both, who have a total score of ≥4 for the above items meet the criteria for primary SS [16]. Level of agreement between these criteria sets has been evaluated as excellent [17], but the real life application of the 2016 ACR/EULAR criteria is still infrequent [18].

### 2.3. Exclusion Criteria

Reviews, abstracts, and sSS were excluded from this review. All articles in which other classification criteria were used were also excluded.

### 2.4. Data Extraction

Two authors of this study (Martinez-Suarez, E. and Manzo, C.) independently reviewed the titles and abstracts of all identified citations. After reviewing the abstracts, data comparisons were conducted to ensure completeness and reliability. The inclusion criteria were independently applied to all identified studies. Differing decisions were resolved by consensus. Full-text versions of potentially relevant papers identified in the initial screening were retrieved. Data concerning study design, source of information, participant characteristics, SS, and assessment of cognitive function were independently extracted.

### 2.5. Assessment of Bias Risk

A subjective assessment of the methodological quality of observational studies was performed by all the authors using the Newcastle-Ottawa Scale, which is a quality assessment tool for non-randomized studies [19]. It uses a “star system” based on three major perspectives: the selection of the study groups (0–4 stars, or 0–5 stars for cross-sectional studies), the comparability of the groups by controlling for important and additional relevant factors (0–2 stars), and the ascertainment of the outcome of interest or exposure (0–3 stars). A total score of 3 or less was considered poor, 4–6 was considered moderate, and 7–10 was considered high quality. Studies scoring 3 or less were excluded from our review. Discrepant opinions between authors were resolved by consensus.

## 3. Results

### 3.1. Description of Included Studies

**Figure 1:**
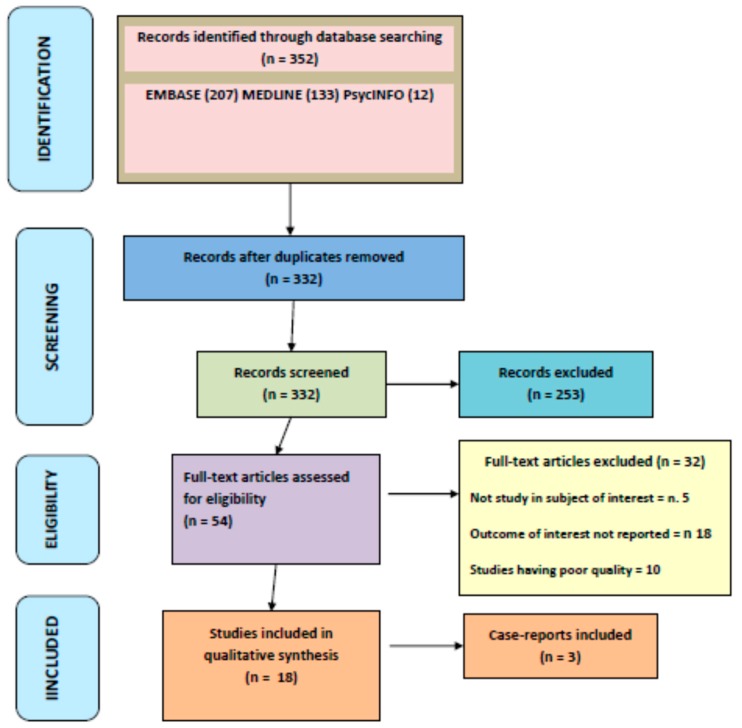
PRISMA flow-chart of the systematic review process.

As reported in Figure 1, the initial search yielded 352 articles, of which 253 articles were excluded based on the title and abstract review. A total of 54 articles underwent a full-length review, and 32 articles were excluded (not a study in subject of interest = 5; studies having poor quality = 10; no outcome of interest = 18). Data were extracted from 18 studies and 3 case-reports, involving a total of 6196 subjects.

The characteristics of studies included in this review are outlined in Table 1.

### 3.2. pSS, Mild Cognitive Impairment (MCI), and Dementia

Six studies appraised the relationships between pSS, MCI, and dementia [22,25,26,27,32,33]. Yoshikawa et al. published a retrospective study and evaluated a cohort of 20 patients with pSS and dementia or cognitive dysfunction in general. Their age ranged from 56 to 92 years. They found that 12 patients were affected by dementia (four by Alzheimer’s type; six by vascular dementia; one by mixed dementia; one by normal pressure hydrocephalus), and eight by MCI (five of them had a vascular MCI). In all patients, brain magnetic resonance imaging (MRI) and single-photon emission computed tomography (SPECT) were performed. A correlation with depression was found, whereas no correlation with other extraglandular manifestations of pSS was observed [26].

In a case-control prospective study, Blanc et al. found that among 25 patients with pSS, 15 (60%) presented with cognitive dysfunction; five of these had dementia. Dementia was diagnosed according to the Diagnostic and Statistical Manual of Mental Disorders, fourth edition, text revision (DSM-IV-TR). According to Petersen’s criteria, 10 of the patients had MCI (five non-amnestic single domain and five amnestic). Their age ranged from 30 to 74 years. The mean duration of cognitive complaints was 6.1 ± 5.6 years. Neuropsychological symptoms came first for six patients. Brain MRI was performed in all patients, and a trend towards a correlation was found between the severity of cognitive impairment and the degree of white matter lesions (WML) with a *p*-value = 0.03. No cases of MCI or dementia were found in the control group [32].

In a case-control study, Dziadkowiak et al. evaluated 30 consecutive patients with pSS (median age of 51 years), and found that cognitive impairment correlated with disease duration and with severity of inflammatory changes [33].

In 2015, Morreale et al. published their data regarding 87 patients with recent (<6 months) diagnosis of pSS and found that 31.1% had cognitive dysfunction compatible with dementia. Brain MRI was performed [25].

In 28 patients with pSS, Tezcan et al. found that 78.8% had cognitive impairment of the frontal- subcortical type and 11% had a severe cognitive impairment. No neuroimaging evaluation was performed. In two patients, there was no active extraglandular manifestation during neuropsychological assessment [22].

More recently, in a population study based on the Taiwan National Health Insurance Research Database (NHIRD), Lin et al. found that, among 4756 patients with pSS, 238 had dementia. However, the type of dementia was not specified. In NHIRD, parameters such as clinical severity or laboratory data are not recorded [27].

In these studies, brain MRI, when performed, mostly showed frontal and subcortical pathological findings. However, in several patients MRI scans were normal.

### 3.3. Fatigue and Cognitive Function in pSS

In pSS patients, fatigue prevalence is between 67% and 85 %, and it can be considered a good indicator of systemic activity [40,41]. Indeed, the assessment of fatigue is present in two of the most important pSS activity scales, namely the “Sjogren’s syndrome disease activity index (SSDAI)” [42,43] and the “Sjogren’s syndrome systemic clinical activity index (SCAI)” [44]. A strong correlation was observed between the presence of subjective cognitive symptoms and fatigue severity, and dysfunction in attention, executive functions, working memory, and verbal memory among objective cognitive tests [45]. Among the studies included in this review, eight also had assessed fatigue [20,22,28,29,30,33,34,36]. The case-control study published in 2016 by Kocer et al. was of great interest; 32 patients with pSS were evaluated through comprehensive neuropsychological tests and neuroimaging. In the other six studies included in this review, neuroimaging was not performed. Fatigue was evaluated by using the Fatigue Severity Scale (FSS). FSS is a nine-item self-administered instrument. The scores for each item range from one to seven, with the lower score indicating less fatigue. A FSS score ≥4 reliably differentiates subjects with fatigue from controls. In this study, no statistically significant differences were observed in terms of all neuropsychological tests, depression, fatigue severity, health state, and daily-life activities between PSS and control cases without depression. These findings indicate the presence of a pattern of cognitive impairment without the negative effects of depression in patients with pSS. Nevertheless, the authors found a correlation between fatigue and cognitive dysfunction, with a *p*-value <0.05 when they were compared with the healthy control group. In particular, a significant negative correlation between the Clock Drawing and the SF-36 was observed. However, they highlighted that fatigue may not affect neuropsychological-tests performance in well-selected patients [29]. In the other included studies, a certain terminological confusion was found. In particular, confusion among fatigue, weakness, and stiffness was particularly frequent.

### 3.4. Laboratory Data and Clinical Manifestations

In the majority of the studies included in this review, correlations between extraglandular manifestations, laboratory data, and cognitive dysfunction were not assessed or not found. Delalande et al.’s study was particularly interesting, because they found that in 47% of patients sicca symptoms were absent [4]. The potential pathogenetic role of antibodies against the NR-2 subtype of N-methyl-D-aspartate (NMDA) receptors was particularly evaluated in the study performed by Lauvsnes et al. The authors found that cognitive dysfunction and depression occurred more frequently in pSS patients with anti-NR2 antibodies than in pSS patients without them. A correlation between anti-NR2 antibodies, cognitive dysfunction, and hippocampus volume was also highlighted in a subgroup of their pSS patients [35].

## 4. Discussion

This systematic review highlighted that cognitive impairment can occur in patients with pSS. Many individuals reported mild cognitive difficulties, often referred to as the so-called “brain fog”. We know that brain fog is a characteristic of fibromyalgia; some investigators have referred to it as fibro-fog [46,47]. A brain fog has been reported in 50 – 80% of fibromyalgic patients [46,47,48]. Is the brain fog of fibromyalgic patients identical to that of patients with pSS? Is there a “Sjo-fog”? In this review, it is postulated and argued that brain fog in pSS is not different from that of fibromyalgia. As in fibromyalgia, its physiological basis in pSS is not clearly understood, but it has been suggested to be multifactorial with pain, depression, sleep, and some drugs being the most important determinants [46,47,48,49]. In clinical practice, the possibility that brain fog can be related to a concomitant or overlapping fibromyalgia must be carefully considered [49,50].

A possible immune-mediated endothelitis has been hypothesized in brain fog in patients with pSS, but this review did not identify studies confirming this hypothesis. This unmet need should be addressed in ad hoc future studies.

Dementia is less commonly described in the literature, with subcortical dementia as the most frequent clinical finding [20,21,22,23,24]. Furthermore, pSS is common in the elderly [51] and older age can be a common factor with AD. In a nation-wide retrospective population study, which was not included in this review because it did not meet the inclusion criteria, a higher prevalence of AD was found in pSS patients older than 70 years than in non-pSS patients, suggesting that pSS should be considered as a risk factor for AD [52]. In another prospective study, recruiting patients with dementia in a memory clinic, patients with pSS accounted for 7.5% of those with cognitive impairment [23].

This systematic review suggests that four important points must be taken into account in clinical practice:

1) Dementia can occur in patients with pSS. With increasing ageing, subcortical dementia is usually due to vascular brain pathology or synucleinopathies (i.e., Parkinson’s disease and Lewy body disease), but it can also be associated with pSS in a small proportion of cases (5%) [8], although the actual causal mechanisms or relationship remains to be elucidated from current available knowledge.

2) An autoimmune-induced dementia is possible in pSS. Autoimmune dementia is, by definition, reversible. It may be misdiagnosed as a primary, degenerative dementia, leading to serious therapeutic errors.

3) Normal findings in brain MRI are frequent in patients with pSS.

4) Cognitive dysfunction can be the first clinical manifestation of pSS.

Hence, it is very important that clinicians look for sicca syndrome or for laboratory data suggestive of pSS when direct observation and neurologic tests detect problems with information processing, attention, memory, or executive function, even if brain MRI is normal or in the presence of a few WML. SPECT assessment should be considered, as it revealed a hypoperfusion in parietal, temporal, and frontal lobes in more than 50% of patients having normal MRI brain when cognitive dysfunction was present. This SPECT pattern was present only in 17% of pSS patients without cognitive dysfunction [20,21,22,23,24,25,26,27,28,29,30,31,32,33,34,35,36,37,38,39,53].

The first observation of a correlation between CNS involvement and laboratory data is in the series of eight patients described by Alexander et al. in 1982, in which a direct pathogenetic role of the antiRo (SSA) antibodies was suggested [54]. However, cognitive dysfunction may occur even in the seronegative forms of pSS [20,54], and this must be taken into account in clinical practice.

NR2 receptors are present in particularly high-density in the hippocampus. Binding of anti-NR2 antibodies to these receptors causes neuronal death through an increase of intracellular levels of calcium. Anti-NR2 antibodies may play a pathogenetic role in cognitive dysfunction in pSS, and correlation between their levels and the severity of cognitive dysfunction have been suggested [32]. However, this hypothesis should be confirmed in multicenter, large studies.

Finally, the roles of cryoglobulinemia and antiphospholipid syndrome were more than anecdotal in this review [18].

Fatigue is a frequent and destabilizing manifestation in patients with pSS and it can influence their cognitive performances. This review identified only one good-quality article that evaluated this relationship, confirming a bidirectional interference between fatigue and cognitive functions [29]. The interference of pain was not always taken into account. For example, testing patients during pSS relapses should favor it. From the review, it is not possible to tease out the influence of fatigue and pain on cognitive functions in patients with pSS.

Our review identified areas that demand further insight. First, some of the included studies did not report the severity of pSS [17,21,23,25,28,29], and few correlations between disease severity and cognitive function were possible. Second, the evaluation of the pathogenetic role of comorbid diseases was often absent [17,21,22,23,24,25]. The lack of information on the type of dementia represented a third critical point in the majority of the included studies [7,20,22,24,25,26,27,28,30,31,32,33,34].

## 5. Conclusions

Cognitive dysfunction can be present in patients with pSS, and, in some cases, it is the first clinical manifestation.

In most cases, a brain fog or MCI are present. Occasionally, pSS can cause an autoimmune dementia that would be reversible by definition. However, the relationship between pSS and degenerative dementias, such as AD, is not clear, even if some investigators have hypothesized that pSS could be a risk factor. In elderly onset SS, older age can be a common risk factor.

When cognitive impairment is diagnosed in a middle-aged patient, a search for sicca syndrome or for laboratory data suggestive of pSS should be considered. However, the possibility that sicca symptoms can be absent in pSS patients with cognitive dysfunction, as well as that cognitive dysfunction can be present in seronegative forms of pSS, must be taken into account.

The routine assessment of cognitive function in patients with pSS seems advisable, and will inform the future studies designed to explore some of the knowledge gaps highlighted by this review.

## Figures and Tables

**Table 1:** Characteristics and main data of the studies included in this review.

	1st Author Year	Study Design	Patients F/M	Cognitive Impairment	Depression	Fatigue	Subset of Cognitive Impairment	Neuro Imaging	Correlation with Extraglandular Manifestations
1	Segal BM, 2012 [20]	Case-control Monocentric data	39 39/0	YES 30% psychomotor processing (DST) verbal reasoning (similarities)	Yes, CES_D 47% vs. 6%	Yes, FSS	Frontal and subcortical brain type	Absent	No
2	Le Guern V, 2009 [21]	Case-control Monocentric data	10 10/0	YES 80% visuospatial abilities executive function	Yes Hamilton depression scale	No	Frontal-lobe-related syndrome	MR and 99m Tc-ECD SPECT	Yes 7/10 patients 1 positive for antiphospholipid serology and obstetric APLS 3 still active
3	Tezcan ME, 2016 [22]	Case-control Monocentric data	28 28/0	YES 78.8% immediate verbal memory Clock drawing test attention and information processing speed verbal learning	Yes	Yes	Frontal and subcortical brain type 3/28 (11%) with serious cognitive dysfunction, 11 (39.3%) with MCI, 8 (28.5%) had moderate cognitive dysfunction	Absent	Yes 2/28 patients: One patient liver and one patient lung involvement Antiganglioside antibody 42.8% No active extraglandular manifestation during neurological assessment
4	Delalande S, 2004 [23]	Retrospective study Monocentric data	82 65/17	YES 13.04% Authors did not systematically screen their patients with pSS for cognitive impairment.	No	No	Not clear In 2 patients, subcortical dementia	Yes Brain MRI	Yes 71/86 in 47% of patients absence of sicca symptoms!
5	Rodrigues DN, 2014 [24]	Case-control Monocentric data	18 18/0	YES Executive functioning Long-term memory tests	Yes Beck Depression Inventory	No	Subcortical brain type	Not reported	No
6	Morreale M, 2015 [25]	Case-control Monocentric data	87 78/9	Yes 31.1% Executive dysfunction Visuospatial disorders Short-term memory deficits	Yes Beck Depression Inventory	Not clear	Not clear Patients with recent diagnosis (<6 months) of pSS	Yes Brain MRI Transcranial Doppler	Yes
7	Yoshikawa K, 2012 [26]	Prospective Study Cohort of patients with dementia or cognitive dysfunction Monocentric data	20 15/5	YES 13 had dementia (4 Alzheimer’s type; 6 vascular dementia; 1 mixed dementia) 7 had mild cognitive impairment	Yes	No	4 Alzheimer’s type 6 vascular dementia 1 mixed dementia 1 normal pressure hydrocephalus 8 mild cognitive impairment: 5 vascular MCI	YES Brain MRI revealing subcortical lesions. Brain SPECT revealing asymmetrical focal hypoperfusion.	No
8	Lin TM, 2018 [27]	Population study Taiwan National Health Insurance Research Database	4756 not possible	YES 238 patients had dementias No information about the type of dementia is present.	No	No	Not specified	Yes	No The NHIRD does not contain such parameters such as clinical severity and laboratory data.
9	Omma A, 2018 [28]	Case-control Multicenter data Cross-sectional study	97/8	YES SF-36 low score for the mental-health subdimension	YES	YES	Not clear	No	Not clear
10	Ye W, 2018 [7]	Cohort study Monocentric data Cross-sectional study	415 10/1 ratio	YES 1.03% 4/415 had cognitive dysfunction	Yes 10 pts had depression	No	Not reported	YES Brain MRI	YES 125/415 pts
11	Koçer B, 2016 [29]	Case-control Monocentric data	32 32/0	YES Low performance in Clock Drawing, COWAT, PASAT, Colourless Word Reading (Stroop1) and Recognizing Colours (Stroop2) Patterns of STROOP test, AVLT immediate verbal memory (A1) and long-term verbal memory (A7) patterns, BJLOT, and in all the patterns of RCFT in PSS patients compared to the healthy control group (*p* < 0.05)	Yes SF (pain) 53.44 Hamilton + in 6.69	Yes	Not reported	YES SPECT (Hypo perfusion in parietal, temporal, and frontal lobes was 56.3%)	No
12	Wouters EJM, 2012 [30]	Case-control Two-centre data	294 273/21	No	Not reported	Yes	Not reported	Absent	No
13	Hartkamp A, 2004 [31]	Case-control Two-centre data	60 60/0	No	Yes MFI; General fatigue and physical fatigue were present in 75%, reduced activity in 63%, and reduced motivation and mental fatigue in 52%	No	Not reported	Absent	Yes Cytokine level above the lower range of detection of the assay used was present in 13% of patients for IL2, 5 (8%) for IL6, 32% for IL10, and 83% for TNFa
14	Blanc F, 2013 [32]	Case-control Monocentric data Prospective data	25 21/4	Yes 60% Five patients had dementia in the PSS group. Ten patients had MCI, five had a non-amnestic single domain, and five an amnestic MCI multi-domain.	No, but two pSS patients took antidepressant drugs at the time of evaluation.	No	Five patients had dementia. 10 patients had MCI, five had a non-amnestic single domain, and five an amnestic MCI multi-domain	Brain MRI. A trend towards a correlation was found between WML and the severity of cognitive dysfunctions	No. All patients had sicca symptoms and in 20 of these abnormal histopathology of the minor salivary glands was found. No correlation was found between the severity of cognitive impairment and duration of pSS.
15	Dziadkowiak E, 2015 [33]	Case-control Monocentric data	30 29/1	No MMSE and CDT normal	Yes Subjective depressed mood >2 w 20%	Yes Subjective chronic fatigue 63%	Not Reported	Absent Endogenous cognitive event-related potentials (CERPs)	Yes Sicca symptoms 96%, lymphocytic infiltration in labial salivary gland 93%: there was a significant prolongation of the latency of P300 and N200 potentials
16	Goodchild CE, 2010 [34]	Cohorts pSS and RA Prospective Multicentric (clinics)	14 14/0	No	No	Yes Mental fatigue 1.74, somatic fatigue 2.83	Not Reported	Absent	Yes Oral sicca 2.92, ocular sicca 2.36 (profile of discomfort)
17	Lauvsnes MB, 2013 [35]	Case-control Monocentric data	66 58/8	No	Yes BDI (30%)	No	Not reported	MRI revealed a smaller hippocampus volume	Yes Anti-NR2 antibodies (were associated with a worse performance in 8 of 10 memory and learning tests); in 20% of pSS patients they were above the cutoff value
18	Hackett K, 2015 [36]	Case-control Cross sectional Multicentric	105 ?	Not reported	Yes Multivariate analysis (with HAD) 0.428	Yes	Not reported	Absent	Yes Dryness VAS (multivariate analysis 0.087)
19	Rosado SN, 2018 [37]	Case report	1 1/0	Yes Neuropsychiatric manifestations associated with severe cognitive dysfunction	Yes	No	Not reported	MRI Normal	Yes AntiRo, antiLa +, Schimer test + Weakly positive for IgM anticardiolipin and anti-B2-glicoprotein 1 with negative IgG.
20	Hayashi Y, 2008 [38]	Case report	1 0/1	Yes 100% Progressive dementia and gait disturbance	No	No	Yes	MRI T1-weighted images with low-intensity lesions, and T2-weighted and FLAIR images with high-intensity lesions in the left frontal white matter, bilateral parietal and left occipital white matter, left thalamus, and right middle cerebellar peduncle, which were not enhanced by Gadolinium	No. Saxon and Shirmer tests Scintigraphy of the parotid and submandibular glands, and lip biopsy that revealed many lymphocytes and plasma cells infiltrating the small salivary glands
21	Hirohata M, 2005 [39]	Case report	1 1/0	Yes 100% Forgetfulness (memory disturbance)	No	No	Yes	MRI, SPECT SPECT perfusion imaging for cerebral blood flow revealed regions of decreased uptake in the parietal lobes. Brain MRI and magnetic resonance angiography (MRA) were normal	

CES-D = Center for Epidemiologic Studies Depression scale; FSS = Fatigue Severity Scale; APLS = Antibodies anti PhoshoLipid syndrome; MCI: Mild Cognitive Impairment; MRI = Magnetic Resonance Imaging; AVLT = Auditory- Verbal Learning Test; BJLOT = Benton Judgment of Line Orientation Test; RCFT = Recognizing Color FunctionTest.

## Supplementary Materials

The following are available online at https://www.mdpi.com/2076-3425/9/4/85/s1. Box 1: Full search terms used together with the results of the searches in MEDLINE.
