# Supplementary material for: Cognitive Function in Primary Sjögren’s Syndrome: A Systematic Review
Source: Brain Sci. 2019 Apr 15;9(4):85. doi: 10.3390/brainsci9040085 (PMC6523842; doi:10.3390/brainsci9040085)
Supplement: Supplementary file 1 [file brainsci-09-00085-s001.pdf]

Supplemental material and methods

Box 1 : <u>***F**ull search terms used together with the results of the searches in MEDLINE*</u>

14 Sep 18 - 12:37

HDAS Export

See full search strategy

Strategy 500941/1

#	Database	Search term	Results
1	Medline	((exp MEMORY DISORDERS/ OR exp MEMORY/ OR ATTENTION/ OR EXECUTIVE FUNCTION/ OR exp COGNITION DISORDERS/ OR exp COGNITION/ OR exp THINKING/ OR exp CONFUSION/ OR exp FATIGUE/ OR PHYSICAL EXERTION/ OR (memor*).ti OR (cognit*).ti OR (attent*).ti OR (confus*).ti OR (fatig*).ti OR (tired*).ti OR (think*).ti OR (thought*).ti OR (impair*).ti) AND ((pss AND (sjoegren OR sjogren)).ti,ab,su OR (primary AND (sjoegren OR sjogren)).ti,ab,su OR ((sjoegren OR sjogren) NOT secondary).ti,ab,su)) [DT 2002-2018]	133

Contents 133 of 133 results on Medline - ((exp MEMORY DISORDERS/ OR exp MEMORY/ OR ATTENTION/ OR EXECUTIVE FUNCTION/ OR exp COGNITION DISORDERS/ OR exp COGNITION/ OR exp THINKING/ OR exp CONFUSION/ OR exp FATIGUE/ OR PHYSICAL EXERTION/ OR (memor*).ti OR (cognit*).ti OR (attent*).ti OR (confus*).ti OR (fatig*).ti OR (tired*).ti OR (think*).ti OR (thought*).ti OR (impair*).ti) AND ((pss AND (sjoegren OR sjogren)).ti,ab,su OR (primary AND (sjoegren OR sjogren)).ti,ab,su OR ((sjoegren OR sjogren) NOT secondary).ti,ab,su)) [DT 2002-2018]

1. The EULAR Sjogren's syndrome patient reported index as an independent determinant of health-related quality of life in primary Sjogren's syndrome patients: in comparison with non-Sjogren's sicca patients.

2. Primary Sjögren's syndrome is associated with significant cognitive dysfunction.

3. Primary Sjögren's syndrome: Extraglandular manifestations and hydroxychloroquine therapy.

4. Autonomic dysfunction in primary Sjogren's syndrome: a prospective cohort analysis of 154 Korean patients.

5. Sicca symptoms are associated with similar fatigue, anxiety, depression, and quality-of-life impairments in patients with and without primary Sjögren's syndrome.

6. Primary Sjogren's syndrome: cognitive symptoms, mood, and cognitive performance.

7. Primary Sjögren's Syndrome White Matter Changes and Cognitive Dysfunction.

8. Measurement of fatigue and discomfort in primary Sjogren's syndrome using a new questionnaire tool.

9. Cognition, depression, fatigue, and quality of life in primary Sjögren's syndrome: correlations.

10. Association between memory B-cells and clinical and immunological features of primary Sjögren's syndrome and Sicca patients.

11. Efficacy and safety of belimumab in primary Sjögren's syndrome: results of the BELISS open-label phase II study.

12. A Transcriptional Signature of Fatigue Derived from Patients with Primary Sjögren's Syndrome.

13. A possible genetic association with chronic fatigue in primary Sjögren's syndrome: a candidate gene study.

14. Abatacept treatment reduces disease activity in early primary Sjögren's syndrome (open-label proof of concept ASAP study).

15. Sjögren's Systemic Clinical Activity Index (SCAI)--a systemic disease activity measure for use in clinical trials in primary Sjögren's syndrome.

16. Sleep disordered breathing in patients with primary Sjögren's syndrome: a group controlled study.

17. Fatigue in primary Sjögren's syndrome--a link to sickness behaviour in animals?

18. Efficacy and safety of rituximab treatment in early primary Sjögren's syndrome: a prospective, multi-center, follow-up study.

19. Upper airway surface tension but not upper airway collapsibility is elevated in primary Sjögren's syndrome.

20. Cerebrospinal Flt3 ligand correlates to tau protein levels in primary Sjögren's syndrome.

21. Fibromyalgia prevalence and associated factors in primary Sjögren's syndrome patients in a large cohort from the Spanish Society of Rheumatology registry (SJOGRENSER).

22. The impact of primary Sjögren's syndrome on female sexual function.

23. A five-year prospective study of fatigue in primary Sjögren's syndrome.

24. A qualitative exploration of physical, mental and ocular fatigue in patients with primary Sjögren's Syndrome.

25. Physical activity and physical activity cognitions are potential factors maintaining fatigue in patients with primary Sjogren's syndrome.

26. Cognitive function and 99mTc-ECD brain SPECT are significantly correlated in patients with primary Sjogren syndrome: a case-control study.

27. Pain, xerostomia, and younger age are major determinants of fatigue in Korean patients with primary Sjögren's syndrome: a cohort study.

28. Development of the Sjögren's Syndrome Responder Index, a data-driven composite endpoint for assessing treatment efficacy.

29. Objectively measured physical activity and its influence on physical capacity and clinical parameters in patients with primary Sjögren's syndrome.

30. Fatigue in primary Sjögren's syndrome is associated with lower levels of proinflammatory cytokines.

31. The assessment of fatigue in primary Sjögren's syndrome.

32. Safety and efficacy of leflunomide in primary Sjögren's syndrome: a phase II pilot study.

33. Do the European League Against Rheumatism (EULAR) Sjögren's syndrome outcome measures correlate with impaired quality of life, fatigue, anxiety and depression in primary Sjögren's syndrome?

34. Impact of pain on cognitive functions in primary Sjögren syndrome with small fiber neuropathy: 10 cases and a literature review.

35. Fatigue in daily life in patients with primary Sjögren's syndrome and systemic lupus erythematosus.

36. Neuropsychiatric syndromes in patients with systemic lupus erythematosus and primary Sjögren syndrome: a comparative population-based study.

37. Variability of fatigue during the day in patients with primary Sjögren's syndrome, systemic lupus erythematosus, and rheumatoid arthritis.

38. Health-related utility values of patients with primary Sjögren's syndrome and its predictors.

39. Fatigue and depression predict physician visits and work disability in women with primary Sjögren's syndrome: results from a cohort study.

40. Primary Sjögren's syndrome in children and adolescents: are proposed diagnostic criteria applicable?

41. Serum dehydroepiandrosterone sulphate levels and laboratory and clinical parameters indicating expression of disease are not associated with fatigue, well-being and functioning in patients with primary Sjögren's syndrome.

42. Serum cytokine levels related to multiple dimensions of fatigue in patients with primary Sjogren's syndrome.

43. Refractory primary Sjögren syndrome successfully treated with bortezomib.

44. Cognitive Dysfunction and Dementia in Primary Sjögren's Syndrome.

45. Regulatory mechanisms for the production of BAFF and IL-6 are impaired in monocytes of patients of primary Sjögren's syndrome.

46. CD4 CD25high regulatory T cells are not impaired in patients with primary Sjögren's syndrome.

47. Physical fatigue characterises patient experience of primary Sjögren's syndrome.

48. Treatment of primary Sjögren syndrome.

49. Psychological comorbidities associated with subclinical atherosclerosis in Greek patients with primary Sjögren's syndrome: a potential contribution of sleep impairment.

50. A short neuropsychological evaluation of patients with primary Sjögren's syndrome.

51. Endogenous event-related potentials in patients with primary Sjögren's syndrome without central nervous system involvement.

52. IL-2 Inhibition of Th17 Generation Rather Than Induction of Treg Cells Is Impaired in Primary Sjögren's Syndrome Patients.

53. Daytime patterning of fatigue and its associations with the previous night's discomfort and poor sleep among women with primary Sjögren's syndrome or rheumatoid arthritis.

54. Primary Sjögren's syndrome in Moroccan patients: characteristics, fatigue and quality of life.

55. Histological improvement in salivary gland along with effector memory Th17-1 cell reduction in a primary Sjogren's syndrome patient with dermatomyositis and diffuse large B-cell lymphoma by R-CHOP therapy.

56. Assessment of fatigue and dryness in primary Sjögren's syndrome: Brazilian version of "Profile of Fatigue and Discomfort - Sicca Symptoms Inventory (short form) (PROFAD-SSI-SF)".

57. Impaired gastric emptying in primary Sjogren's syndrome.

58. B7-H4 deficiency in salivary gland of patients with primary Sjögren's syndrome impairs the regulatory effect on T cells.

59. Current and potential treatments for primary Sjögren's syndrome.

60. Epratuzumab (humanised anti-CD22 antibody) in primary Sjögren's syndrome: an open-label phase I/II study.

61. Abnormal B cell differentiation in primary Sjögren's syndrome results in a depressed percentage of circulating memory B cells and elevated levels of soluble CD27 that correlate with Serum IgG concentration.

62. Effects of hydroxychloroquine on symptomatic improvement in primary Sjögren syndrome: the JOQUER randomized clinical trial.

63. Assessment of fatigue in primary Sjögren's syndrome: the Swedish version of the Profile of Fatigue.

64. Low number of memory B cells in the salivary glands of patients with primary Sjögren's syndrome.

65. Primary Sjogrens syndrome is associated with impaired autonomic response to orthostasis and sympathetic failure.

66. Psychological and somatic predictors of perceived and measured ocular dryness of patients with primary Sjögren's syndrome.

67. Randomized Controlled Trial of Rituximab and Cost-Effectiveness Analysis in Treating Fatigue and Oral Dryness in Primary Sjögren's Syndrome.

68. Severe Health-Related Quality of Life Impairment in Active Primary Sjögren's Syndrome and Patient-Reported Outcomes: Data From a Large Therapeutic Trial.

69. White matter abnormalities in primary Sjögren syndrome.

70. Impaired functional status in primary Sjögren's syndrome.

71. [Treatment of primary Sjögren syndrome].

72. The minimally important difference (MID) for patient-reported outcomes including pain, fatigue, sleep and the health assessment questionnaire disability index (HAQ-DI) in primary Sjögren's syndrome.

73. Memory dysfunction in primary Sjögren's syndrome is associated with anti-NR2 antibodies.

74. Fatigue and widespread pain in systemic lupus erythematosus and Sjögren's syndrome: symptoms of the inflammatory disease or associated fibromyalgia?

75. EULAR Sjogren's Syndrome Patient Reported Index (ESSPRI): development of a consensus patient index for primary Sjogren's syndrome.

76. Physical capacity in women with primary Sjögren's syndrome: a controlled study.

77. Fatigue in Primary Sjögren's Syndrome: Clinical, Laboratory, Psychometric, and Biologic Associations.

78. Subjective and Objective Measures of Dryness Symptoms in Primary Sjögren's Syndrome: Capturing the Discrepancy.

79. Classification criteria and treatment modalities in primary Sjögren's syndrome.

80. Reduction of fatigue in Sjögren syndrome with rituximab: results of a randomised, double-blind, placebo-controlled pilot study.

81. Prevalence, severity, and predictors of fatigue in subjects with primary Sjögren's syndrome.

82. Dehydroepiandrosterone (DHEA) substitution treatment for severe fatigue in DHEA-deficient patients with primary Sjögren's syndrome.

83. Epigenome-wide DNA methylation patterns associated with fatigue in primary Sjögren's syndrome.

84. Comparison of the performance of the different classification criteria for primary Sjögren's syndrome: a prospective cohort study.

85. Magnetic resonance abnormalities associated with cognitive dysfunction in primary Sjögren syndrome.

86. Quality of life and psychological status in patients with primary Sjögren's syndrome and sicca symptoms without autoimmune features.

87. Proposed core set of outcome measures in patients with primary Sjögren's syndrome: 5 year follow up.

88. Primary Sjögren's syndrome: fatigue is an ever-present, fluctuating, and uncontrollable lack of energy.

89. Is there progressive cognitive dysfunction in Sjögren Syndrome? A preliminary study.

90. Validation of EULAR primary Sjögren's syndrome disease activity (ESSDAI) and patient indexes (ESSPRI).

91. Fatigue and blood pressure in primary Sjogren's syndrome.

92. [Clinicopathological Features and Treatment of Renal Impair in Primary Sjögren Syndrome].

93. Reversible cortical lesions in primary Sjögren's syndrome presenting with meningoencephalitis as an initial manifestation.

94. Primary Sjögren's syndrome is characterized by distinct phenotypic and transcriptional profiles of IgD+ unswitched memory B cells.

95. Heat shock proteins and chronic fatigue in primary Sjögren's syndrome.

96. Abnormalities in peripheral B cell memory of patients with primary Sjögren's syndrome.

97. Subclinical atherosclerosis and impaired bone health in patients with primary Sjogren's syndrome: prevalence, clinical and laboratory associations.

98. Memory B-cell aggregates in skin biopsy are diagnostic for primary Sjögren's syndrome.

99. 2016 American College of Rheumatology/European League Against Rheumatism classification criteria for primary Sjögren's syndrome: A consensus and data-driven methodology involving three international patient cohorts.

100. Characterization of peripheral natural killer cells in primary Sjögren's syndrome: impaired NK cell activity and low NK cell number.

101. Monocytes from Sjögren's syndrome patients display increased vasoactive intestinal peptide receptor 2 expression and impaired apoptotic cell phagocytosis.

102. Treatment Guidelines for Rheumatologic Manifestations of Sjögren's Syndrome: Use of Biologic Agents, Management of Fatigue, and Inflammatory Musculoskeletal Pain.

103. Fatigue in Sjogren's syndrome: relationship with fibromyalgia, clinical and biologic features.

104. Autoantigen-specific memory B cells in primary Sjögren's syndrome.

105. Impaired clearance of early apoptotic cells mediated by inhibitory IgG antibodies in patients with primary Sjögren's syndrome.

106. Functional impairment of the arterial wall in primary Sjögren's syndrome: combined action of immunologic and inflammatory factors.

107. Sleepiness or fatigue? Can we detect treatable causes of tiredness in primary Sjögren's syndrome?

108. Pain in Sjögren's syndrome.

109. Primary Sjögren's syndrome.

110. Patient-reported outcomes including fatigue in primary Sjögren's syndrome.

111. Gammalinolenic acid treatment of fatigue associated with primary Sjögren's syndrome.

112. Primary Sjogren's syndrome: too dry and too tired.

113. Interleukin-1 inhibition and fatigue in primary Sjögren's syndrome--a double blind, randomised clinical trial.

114. Effects of exercise on aerobic capacity and fatigue in women with primary Sjogren's syndrome.

115. It's more than dryness and fatigue: The patient perspective on health-related quality of life in Primary Sjögren's Syndrome - A qualitative study.

116. Patient-reported outcomes in primary Sjogren's syndrome: comparison of the long and short versions of the Profile of Fatigue and Discomfort--Sicca Symptoms Inventory.

117. Effect of dehydroepiandrosterone administration on fatigue, well-being, and functioning in women with primary Sjögren syndrome: a randomised controlled trial.

118. Calprotectin (S100A8/A9), S100A12, and EDTA-resistant S100A12 complexes (ERAC) in primary Sjögren's syndrome.

119. Autonomic nervous dysfunction development in patients with primary Sjogren's syndrome: a follow-up study.

120. Increased severity of lower urinary tract symptoms and daytime somnolence in primary Sjögren's syndrome.

121. Regular physical activity is associated with lower levels of ESSPRI and other favourable patient-reported outcomes in patients with primary Sjögren's syndrome.

122. Disruption of brain white matter microstructure in primary Sjögren's syndrome: evidence from diffusion tensor imaging.

123. [Epidemiology of primary Sjörgren's syndrome].

124. Validation of the ACR-EULAR criteria for primary Sjögren's syndrome in a Dutch prospective diagnostic cohort.

125. Anti-class a scavenger receptor autoantibodies from systemic lupus erythematosus patients impair phagocytic clearance of apoptotic cells by macrophages in vitro.

126. What do we know about memory B cells in primary Sjögren's syndrome?

127. Management of symptom complexes in primary biliary cholangitis.

128. Prevalence of Sjögren's syndrome with dementia in a memory clinic.

129. Impaired degradation and aberrant phagocytosis of necrotic cell debris in the peripheral blood of patients with primary Sjögren's syndrome.

130. A significantly impaired natural killer cell activity due to a low activity on a per-cell basis in rheumatoid arthritis.

131. Neutrophilic Alveolitis and High Serum Pro-Brain Natriuretic Peptide Level may be Indicators of Pulmonary Functional Impairment in Connective Tissue Disorders.

132. Reduction of Glucocorticoid Receptor Function in Chronic Fatigue Syndrome.

133. Tissue resident memory CD8+ T cells mediate salivary gland damage in a murine model of Sjögren's syndrome.
